# The lncRNA *ANRIL* is down-regulated in peripheral blood of patients with periodontitis

**DOI:** 10.1016/j.ncrna.2020.04.001

**Published:** 2020-04-17

**Authors:** Leila Gholami, Soudeh Ghafouri-Fard, Sara Mirzajani, Shahram Arsang-Jang, Mohammad Taheri, Zahra Dehbani, Safoora Dehghani, Behzad Houshmand, Reza Amid, Arezou Sayad, Bahareh Shams

**Affiliations:** a Dental Research Center, Research Institute for Dental Sciences, Dental School, Shahid Beheshti University of Medical Sciences, Tehran, Iran; b Department of Periodontics, School of Dentistry, Hamadan University of Medical Sciences, Hamadan, Iran; c Urogenital Stem Cell Research Center, Shahid Beheshti University of Medical Sciences, Tehran, Iran.; d Pediatric Cell Therapy Research Center, Tehran University of Medical Sciences, Tehran, Iran; e Department of Biostatistics and Epidemiology, Cancer Gene Therapy Research Center, Faculty of Medicine, Zanjan University of Medical Sciences, Zanjan, Iran; f Department of Periodontics, School of Dentistry, Shahid Beheshti University of Medical Sciences, Tehran, Iran

**Keywords:** Periodontitis, ANRIL, MALAT1, lncRNA

## Abstract

Long non-coding RNAs (lncRNAs) have crucial roles in lncRNAs in periodontal development and disorders of this tissue. A number of lncRNAs especially those regulating immune responses contribute in the pathophysiology of periodontitis. In the current case-control study, we assessed expression levels of two immune response-related lncRNAs namely the antisense non-coding RNA in the INK4 locus (*ANRIL*) and metastasis-associated lung adenocarcinoma transcript 1 (*MALAT1*) in gingival tissues and blood samples of patients with periodontitis and healthy subjects. Expression of *ANRIL* was significantly lower in peripheral blood of patients compared with controls (Posterior Beta RE = -1.734, P value = 0.035). However, when diving study participants based on their gender, no significant difference was found between patients and sex-matched controls. Expression of this lncRNA was not different between periodontitis tissues and normal tissues. Expression of *MALAT1* was not different between samples obtained from cases and controls. Tissue or blood expressions of *ANRIL* or *MALAT1* were not correlated with age of either patients or controls. There were significant correlations between expression levels of *ANRIL* and *MALAT1* in gingival tissues both in cases (r = 0.62, P < 0.0001) and in controls (r = 0.37, P < 0.0001). However, blood levels of these lncRNAs were not correlated with each other either in cases or in controls. Most notably, there was no significant correlation between expression levels of these lncRNAs in gingival tissues and in the blood of study participants. The current study indicates dysregulation of *ANRIL* in the peripheral blood of patients with periodontitis in spite of its normal levels in gingival tissues which might reflect disturbance in systemic immune responses in these patients.

## Highlights

• Expression of *ANRIL* was significantly lower in peripheral blood of patients with periodontitis compared with controls.
• Expression of *ANRIL* was not different between periodontitis tissues and normal tissues.
• Expression of *MALAT1* was not different between samples obtained from cases and controls.
• Tissue or blood expressions of *ANRIL* or *MALAT1* were not correlated with age.

## 1 Introduction

Periodontal disease is a common multifactorial disease that affects nearly all ages, with some groups being more susceptible. The main risk factors are smoking, poor dental care, systemic diseases such as diabetes, certain drugs, age and stress. Besides, genetic factors are regarded as contributing factors in its pathogenesis. Based on its high prevalence, periodontal disease is regarded as a public health problem [1]. The induction of inflammatory reaction to bacteria in the dental biofilm is the main pathogenic event in the periodontitis. Although certain microorganisms are linked with the progressive forms of this disorder; the existence of these microbes in persons with no sign of periodontitis implies that periodontitis is caused as the consequence of the inflammatory responses, not the sole existence of the microorganisms [2]. Regulation of immune responses is a complicated process in which several coding and non-coding genes participate [3]. Among non-coding RNAs whose roles in the pathogenesis of immune-related disorders have been identified are metastasis associated lung adenocarcinoma transcript 1 (*MALAT1*) [4,5] and antisense non-coding RNA in the INK4 locus (*ANRIL*) [6]. *MALAT1* has been shown to be over-expressed patients with systemic lupus erythematous (SLE) patients [4]. Notably, *MALAT1* knock down considerably decreased the expression of IL-21 in primary monocytes of these patients. Additional studies have shown that *MALAT1* role in the pathogenesis of SLE is exerted through regulation of SIRT1 signaling [4], a pathway which is probably important in the reactive oxygen species homeostasis in the process of development of periodontitis [7]. Moreover, *MALAT1* has been upregulated in response to lipopolysaccharide (LPS) [8]. Meanwhile, LPS has a pivotal role in the pathophysiology of periodontitis [9] in a way that severe periodontitis stimulates macrophage functions through this substance [10]. Expression of *MALAT1* has been up-regulated in primary human gingival fibroblasts obtained from patients with periodontitis compared with controls. This lncRNA also enhances expression of inflammatory cytokines through sponging miR‐20a and releasing toll like receptor 4 [11].

*ANRIL* has a prominent role in the pathogenesis of immune-related disorders including coronary artery disease (CAD) [12], type 2 diabetes [13] and cancers [14] as revealed by genome wide association studies. Most notably, the genomic locus for this lncRNA has been identified as a risk locus for periodontitis by various research groups [[15], [16], [17]]. This lncRNA modulates immune response through interaction with the Yin Yang 1 protein [18], a transcription repressor which contribute in the regulation of immune reactions [19]. Certain polymorphisms within *ANRIL* have been associated with levels of C reactive protein (CRP) in CAD patients. Meanwhile, levels of this inflammation mediator have been correlated with severe periodontitis in these patients [20]. Moreover, expression of this lncRNA has been induced in gingival tissues following bacterial infection [21].

Although the role of lncRNAs in the pathophysiology of periodontitis has been uncovered [22], data regarding expression pattern of *ANRIL* or *MALAT1* in gingival tissues or peripheral blood of patients with periodontitis is scarce. Thus, in the current study, we investigated expression of these lncRNAs in these two sets of samples obtained from patients with periodontitis and healthy subjects.

## 2 Material and methods

### 2.1 Enrolled individuals

Tissue samples were obtained during surgical procedure. Cases had the following criteria: chronic periodontitis (Stage II to IV) with at least two remaining periodontal pockets in each sextant after nonsurgical periodontal treatment, probing depth of 5 mm or greater, bleeding on probing (BOP), and at least 3 mm of attachment loss needing surgical periodontal treatment [23]. Moreover, they were older than 18 years and had at least 16 teeth. Exclusion criteria were smoking, systemic diseases, history of consumption of antibiotic or anti-inflammatory drugs 3 months prior to surgical procedures, pregnancy and breastfeeding. Diagnosis of periodontitis was based on the clinical and radiographic examinations performed by a periodontist. Control samples were obtained from BOP sites of patients who underwent crown lengthening. The sites were examined by a periodontist and sites with no BOP and less than 3 mm probing depths were included. The study protocol was approved by ethical committee of Shahid Beheshti University of Medical Sciences (Ethic Code: IR.SBMU.DRC.REC.1398.086).

### 2.2 Expression assays

Total RNA was extracted from both tissue and blood specimens by using Hybrid-RTM blood RNA extraction kit (GeneAll, Seoul, South Korea) according to the protocol provided by the company. The, cDNA was produced from RNA using the OneStep RT-PCR Series Kit (BioFact™, Seoul, South Korea). Relative expressions (RE) of *ANRIL* and *MALAT1* were measured in all specimens using the RealQ Plus 2x PCR Master Mix Green Without ROX™ PCR Master Mix (Ampliqon, Odense, Denmark). Reactions were conducted in StepOnePlus™ RealTime PCR equipment (Applied Biosystems, Foster city, CA, USA) in duplicate. *B2M* gene was used as normalizer. Table 1 shows the sequences of primers and length of PCR products.

**Table 1:** Sequences of primers and length of PCR products.

lncRNA	Primer	Sequence	Product length
*ANRIL*	Forward Reverse	TGCTCTATCCGCCAATCAGG GCGTGCAGCGGTTTAGGTTT	108 bp
*MALAT1*	Forward Reverse	GACGGAGGTTGAGATGAAGC ATTCGGGGCTCTGTAGTCCT	84 bp
*B2M*	Forward Reverse	AGATGAGTATGCCTGCCGTG GCGGCATCTTCAAACCTCCA	105 bp

### 2.3 Statistical methods

Transcript quantities of *ANRIL* and *MALAT1* were compared between periodontitis patients and healthy subjects using Bayesian regression model. The effects of independent variables were adjusted. Statistical analyses were performed in R 3.6.2 software, Rstan, ggplot 2 & non-parametric quantile regression packages. Bootstrap method and 100 iteration methods were used. Correlations between expressions of lncRNAs were evaluated through calculation of Spearman correlation coefficients.

## 3 Results

### 3.1 General characteristics of periodontitis patients and controls

The study included tissue samples from 30 patients with periodontitis (19 females, 11 males) and 30 controls (14 females, 16 males). We also gathered blood samples from 23 patients and 18 healthy controls. Table 2 summarizes the demographic data of periodontitis patients and controls.

**Table 2:** General data of periodontitis patients and controls.

		Patients	Healthy controls
Total Number of Tissues		30	30
Sex	Female	19	14
	Male	11	16
Age (mean ± SD)		41.28 ± 2.7	38.8 ± 1.9
Total Number of Blood Samples		23	18
Sex	Female	15	11
	Male	8	7
Age (mean ± SD)		39.65 ± 3.1	37.91 ± 2.8

### 3.2 Expression assays

**Fig. 1:**
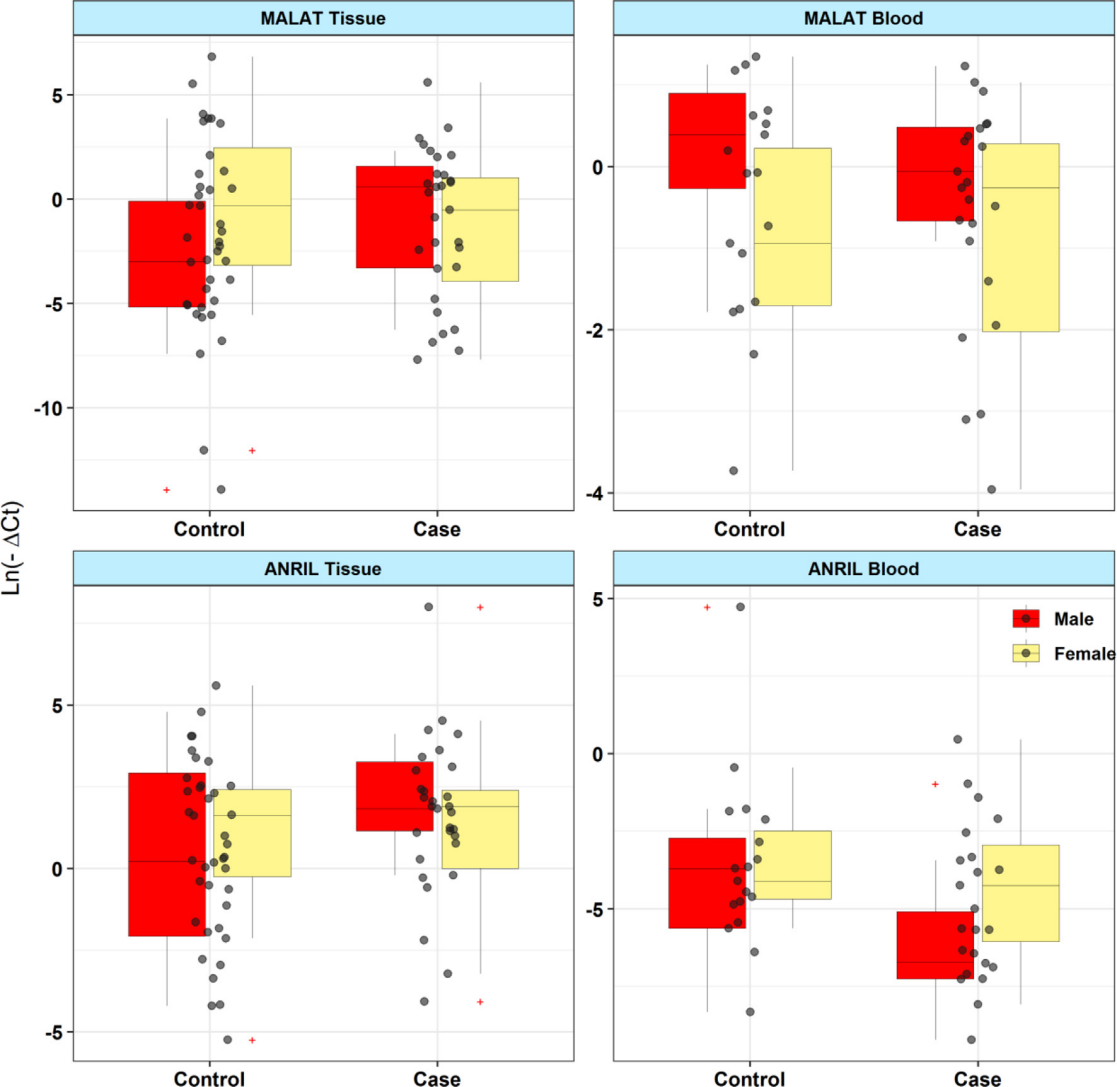
Relative expression (RE) of *ANRIL* and *MALAT1* in tissue and blood samples obtained from patients and controls.

Fig. 1 shows relative expression (RE) of *ANRIL* and *MALAT1* in tissue and blood samples obtained from patients and controls.

Expression of *ANRIL* was significantly lower in peripheral blood of patients compared with controls (Posterior Beta of RE = -1.734, P value = 0.035). However, when diving study participants based on their gender, no significant difference was found between patients and sex-matched controls (Posterior Beta of RE [95% CI] = -2.537 [-7.43, 2.59], P value = 0.043 for males; Posterior Beta of RE [95% CI] = -1.62 [-3.44, 0.45], P value = 0.209 for females). Expression of this lncRNA was not different between periodontitis tissues and normal tissues (Posterior Beta of RE = 0.549, P value = 0.12). This pattern was also seen among male subgroups (Posterior Beta of RE = 1.256, P value = 0.504) and among female subgroups (Posterior Beta of RE = 0.4, P value = 0.568). There was no correlation between tissue/blood levels of this lncRNA and age in either subgroups (Table 3).

**Table 3:** Relative expression of *ANRIL* in tissues and blood specimens of periodontitis patients compared with controls (RE: relative expression, SE: standard error, CrI: credible interval).

Tissue						Blood			
Parameters and groups	Variable	Posterior Beta of RE	SE	P-Value	95% CrI for RE	Posterior Beta of RE	SE	P-Value	95% CrI for RE
Total	Case/Control	0.549	0.684	0.12	[-0.71, 1.98]	**−1.734**	**0.871**	**0.035**	**[-3.46, -0.03]**
	Gender (F/M)	0.195	0.673	0.522	[-1.22, 1.42]	1.282	0.815	0.482	[-0.29, 2.92]
	Age (year)	0.021	0.032	0.635	[-0.04, 0.08]	0.056	0.073	0.255	[-0.08, 0.2]
Male	Case/Control	1.256	1.272	0.504	[-1.07, 3.87]	−2.537	2.538	0.043	[-7.43, 2.59]
	Age (year)	0.074	0.054	0.141	[-0.03, 0.18]	0.051	0.13	0.33	[-0.19, 0.31]
Female	Case/Control	0.4	0.778	0.568	[-1.06, 2.01]	−1.62	0.995	0.209	[-3.44, 0.45]
	Age (year)	−0.024	0.039	0.287	[-0.11, 0.05]	0.119	0.082	0.109	[-0.04, 0.28]

Expression of *MALAT1* was not different between blood/gingival tissues of patients compared with controls (Posterior Beta of RE = 0.939, P value = 0.404 for tissues; Posterior Beta of RE = 0.055, P value = 0.605 for blood samples). Assessment of its expression levels in sex-based groups revealed no significant difference either between male patient and male controls (Posterior Beta of RE = 2.436, P value = 0.116 for tissues; Posterior Beta of RE = 0.595, P value = 0.64 for blood samples) or between female patients and female controls (Posterior Beta of RE = -0.161, P value = 0.88 for tissues; Posterior Beta of RE = 0.043, P value = 0.64 for blood samples) (Table 4).

**Table 4:** Relative expression of *MALAT1* in tissues and blood specimens of periodontitis patients compared with controls (RE: relative expression, SE: standard error, CrI: credible interval).

Tissue						Blood			
Parameters and groups	Variable	Posterior Beta of RE	SE	P-Value	95% CrI for RE	Posterior Beta of RE	SE	P-Value	95% CrI for RE
Total	Case/Control	0.939	1.264	0.404	[-1.6, 3.4]	0.055	0.494	0.605	[-0.89, 1.04]
	Gender (F/M)	1.651	1.067	0.038	[-0.46, 3.64]	−0.463	0.376	0.091	[-1.27, 0.24]
	Age (year)	−0.006	0.061	0.608	[-0.12, 0.11]	−0.017	0.028	0.676	[-0.07, 0.04]
Male	Case/Control	2.436	1.887	0.116	[-1.33, 5.98]	0.595	1.2	0.64	[-1.67, 2.98]
	Age (year)	−0.018	0.079	0.734	[-0.17, 0.15]	−0.046	0.055	0.782	[-0.15, 0.06]
Female	Case/Control	−0.161	1.589	0.88	[-3.2, 2.96]	0.043	0.654	0.64	[-1.26, 1.37]
	Age (year)	−0.034	0.078	0.798	[-0.18, 0.12]	−0.001	0.049	0.341	[-0.1, 0.1]

### 3.3 Correlation between expression levels of *ANRIL* and *MALAT1* lncRNAs and age of enrolled individuals

**Fig. 2:**
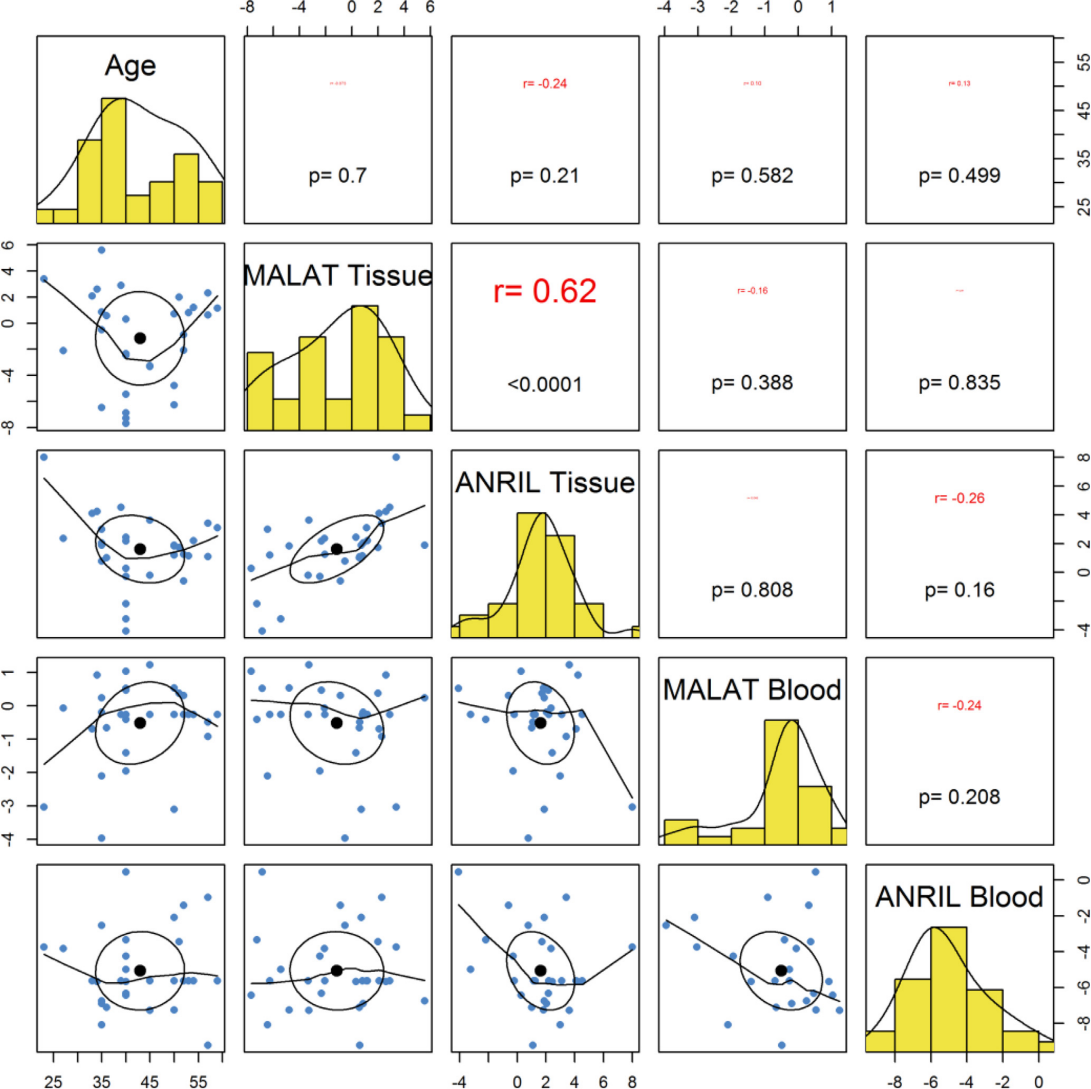
Correlation between expression levels of lncRNAs and age of periodontitis patients.

**Fig. 3:**
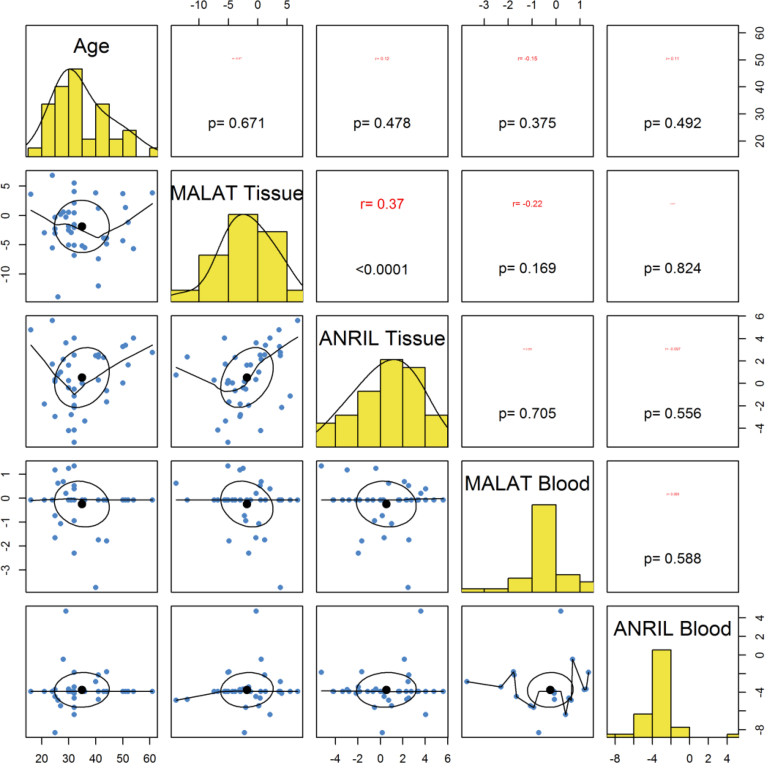
Correlation between expression levels of lncRNAs and age of controls.

Tissue or blood expressions of *ANRIL* or *MALAT1* were not correlated with age of either patients or controls (Fig. 2, Fig. 3). There were significant correlations between expression levels of *ANRIL* and *MALAT1* in gingival tissues both in cases (r = 0.62, P < 0.0001) and in controls (r = 0.37, P < 0.0001). However, blood levels of these lncRNAs were not correlated with each other either in cases (r = -0.24, P = 0.208) or in controls (P = 0.588). Most notably, there was no significant correlation between expression levels of these lncRNAs in gingival tissues and in the blood of study participants (P = 0.556 for *ANRIL* and P = 0.388 for *MALAT1*) (Fig. 2, Fig. 3).

## 4 Discussion

In the present study, we investigated expression of two lncRNAs in peripheral blood and affected gingival tissues of patients with periodontitis and healthy subjects. Expression of *ANRIL* was significantly lower in peripheral blood of patients compared with controls. However, expression of this lncRNA was not different between periodontitis tissues and normal tissues. This lncRNA has been recognized as a shared locus for both periodontitis and CAD [17]. Consistent with the mentioned results, a recent study demonstrated down-regulation of *ANRIL* in peripheral blood of CAD patients compared with healthy subjects [24]. Another study showed down-regulation of the main endothelial cell-associated transcript of *ANRIL* in CAD coronary arteries compared with non-CAD arteries, thus indicating the protective role for *ANRIL* against CAD [25]. Our present results also imply a protective role for this lncRNA against periodontitis, a disorder which has been linked with CAD in both genetic risks [17] and mechanisms [9]. Furthermore, the rs1333048 polymorphism within *ANRIL* has been associated with higher plasma levels of C reactive protein (CRP) in patients with periodontitis [26]. Notably, CRP is regarded a marker for CAD as well [27].

Expression of *MALAT1* was not different between tissue or blood samples obtained from cases and controls. This lncRNA has been demonstrated to modulate immune responses [3]. Its silencing has enhanced expression of TNF-α and IL-6 cytokines [8]. In addition, *MALAT1* has an inhibitory effect on NF-κB signaling, pathway that regulates several aspects of inflammatory responses [8]. This lncRNA also modulates macrophage activation to induce differentiation of M2 macrophages [28]. Notably, *in silico* analysis of miRNA and mRNA expression profiles has shown the role of *MALAT1* in construction of the lncRNA-associated competing endogenous RNA network of periodontitis [22]. Our result was not consistent with the supposed role of this lncRNA in the pathogenesis of immune-related disorders. This might indicate different roles of this lncRNA in the pathogenesis of different immune-related disorders.

Besides, tissue or blood expressions of *ANRIL* or *MALAT1* were not correlated with age of either patients or controls. Thus, if future studies reveal the biomarker role for *ANRIL*, as expression of this lncRNA is not influenced by the age, this biomarker can be used for follow-up of patients during long periods of time.

There were significant correlations between expression levels of *ANRIL* and *MALAT1* in gingival tissues both in cases and in controls. However, blood levels of these lncRNAs were not correlated with each other either in cases or in controls. These observations may indicate the presence of tissue-specific regulatory mechanisms or interaction networks between these lncRNAs which should be explored in future studies. Consistent with this hypothesis, there was no significant correlation between expression levels of these lncRNAs in gingival tissues and in the blood of study participants.

The current study indicates dysregulation of *ANRIL* in the peripheral blood of patients with periodontitis in spite of its normal levels in gingival tissues which might reflect disturbance in systemic immune responses in these patients. Most notably, this finding is in line with the role of *ANRIL* in the pathogenesis of CAD, a disease which is genetically and mechanistically related with periodontitis.

## CRediT authorship contribution statement

**Leila Gholami:** Data curation. **Soudeh Ghafouri-Fard:** Writing - original draft. **Sara Mirzajani:** Methodology. **Shahram Arsang-Jang:** Formal analysis, Data curation. **Mohammad Taheri:** Supervision. **Zahra Dehbani:** Formal analysis, Data curation. **Safoora Dehghani:** Methodology. **Behzad Houshmand:** Data curation. **Reza Amid:** Data curation. **Arezou Sayad:** Supervision. **Bahareh Shams:** Data curation.

## Declaration of competing interest

The authors declare they have no conflict of interest.

## References

[1] Nazir M.A. (2017). Prevalence of periodontal disease, its association with systemic diseases and prevention. Int. J. Health Sci..

[2] Cekici A., Kantarci A., Hasturk H., Van Dyke T.E. (2000). Inflammatory and immune pathways in the pathogenesis of periodontal disease. Periodontol.

[3] Hadjicharalambous M.R., Lindsay M.A. (2019). Long non-coding RNAs and the innate immune response. Noncoding RNA.

[4] Yang H., Liang N., Wang M., Fei Y., Sun J., Li Z. (2017). Long noncoding RNA MALAT-1 is a novel inflammatory regulator in human systemic lupus erythematosus. Oncotarget.

[5] Ghafouri-Fard S., Ashrafi Hafez A., Taheri M. (2020). Metastasis Associated Lung Adenocarcinoma Transcript 1: an update on expression pattern and functions in carcinogenesis. Exp. Mol. Pathol..

[6] Mirza A.H., Berthelsen C.H., Seemann S.E., Pan X., Frederiksen K.S., Vilien M. (2015). Transcriptomic landscape of lncRNAs in inflammatory bowel disease. Genome Med..

[7] Liu C., Mo L., Niu Y., Li X., Zhou X., Xu X. (2017). The role of reactive oxygen species and autophagy in periodontitis and their potential linkage. Front. Physiol..

[8] Zhao G., Su Z., Song D., Mao Y., Mao X. (2016). The long noncoding RNA MALAT 1 regulates the lipopolysaccharide‐induced inflammatory response through its interaction with NF‐κB. FEBS Lett..

[9] Liljestrand J.M., Paju S., Buhlin K., Persson G.R., Sarna S., Nieminen M.S. (2017). Lipopolysaccharide, a possible molecular mediator between periodontitis and coronary artery disease. J. Clin. Periodontol..

[10] Pussinen P.J., Vilkuna-Rautiainen T., Alfthan G., Palosuo T., Jauhiainen M., Sundvall J. (2004). Severe periodontitis enhances macrophage activation via increased serum lipopolysaccharide. Arterioscler. Thromb. Vasc. Biol..

[11] Li J., Wang M., Song L., Wang X., Lai W., Jiang S. (2019). Lnc RNA MALAT 1 regulates inflammatory cytokine production in lipopolysaccharide‐stimulated human gingival fibroblasts through sponging miR‐20a and activating TLR 4 pathway. J. Periodontal. Res..

[12] McPherson R., Pertsemlidis A., Kavaslar N., Stewart A., Roberts R., Cox D.R. (2007). A common allele on chromosome 9 associated with coronary heart disease. Science.

[13] Zeggini E., Weedon M.N., Lindgren C.M., Frayling T.M., Elliott K.S., Lango H. (2007). Replication of genome-wide association signals in UK samples reveals risk loci for type 2 diabetes. Science.

[14] Taheri M., Ghafouri-Fard S. (2018). Antisense non-coding RNA in the INK4 locus (ANRIL) in human cancers. Int. J. Canc. Manag..

[15] Schaefer A.S., Bochenek G., Manke T., Nothnagel M., Graetz C., Thien A. (2013). Validation of reported genetic risk factors for periodontitis in a large-scale replication study. J. Clin. Periodontol..

[16] Schaefer A.S., Richter G.M., Dommisch H., Reinartz M., Nothnagel M., Noack B. (2011). CDKN2BAS is associated with periodontitis in different European populations and is activated by bacterial infection. J. Med. Genet..

[17] Schaefer A.S., Richter G.M., Groessner-Schreiber B., Noack B., Nothnagel M., El Mokhtari N.E. (2009). Identification of a shared genetic susceptibility locus for coronary heart disease and periodontitis. PLoS Genet..

[18] Zhou X., Han X., Wittfeldt A., Sun J., Liu C., Wang X. (2016). Long non-coding RNA ANRIL regulates inflammatory responses as a novel component of NF-κB pathway. RNA Biol..

[19] Lin J., He Y., Chen J., Zeng Z., Yang B., Ou Q. (2017). A critical role of transcription factor YY1 in rheumatoid arthritis by regulation of interleukin-6. J. Autoimmun..

[20] Reichert S., Seitter L., Schaller H.-G., Schlitt A., Schulz S. (2020). ANRIL polymorphisms (rs1333049 and rs3217992) in relation to plasma CRP levels among in-patients with CHD. Cytokine.

[21] Schaefer A.S., Richter G.M., Dommisch H., Reinartz M., Nothnagel M., Noack B. (2011). CDKN2BAS is associated with periodontitis in different European populations and is activated by bacterial infection. J. Med. Genet..

[22] Li S., Liu X., Li H., Pan H., Acharya A., Deng Y. (2018). Integrated analysis of long noncoding RNA-associated competing endogenous RNA network in periodontitis. J. Periodontal. Res..

[23] Sayad A., Taheri M., Sadeghpour S., Omrani M.D., Shams B., Mirzajani S. (2020). Exploring the role of long non-coding RNAs in periodontitis. Meta Gene.

[24] Ebadi N., Ghafouri-Fard S., Taheri M., Arsang-Jang S., Omrani M.D. (2020). Expression analysis of inflammatory response-associated genes in coronary artery disease. Arch. Physiol. Biochem..

[25] Cho H., Shen G.Q., Wang X., Wang F., Archacki S., Li Y. (2019). Long noncoding RNA ANRIL regulates endothelial cell activities associated with coronary artery disease by up-regulating CLIP1, EZR, and LYVE1 genes. J. Biol. Chem..

[26] Teeuw W.J., Laine M.L., Bizzarro S., Loos B.G. (2015). A lead ANRIL polymorphism is associated with elevated CRP levels in periodontitis: a pilot case-control study. PloS One.

[27] Auer J., Berent R., Lassnig E., Eber B. (2002). C-reactive protein and coronary artery disease. Jpn. Heart J..

[28] Cui H., Banerjee S., Guo S., Xie N., Ge J., Jiang D. (2019). Long noncoding RNA Malat1 regulates differential activation of macrophages and response to lung injury. JCI insight.

